# Inheritance genetics of the trait vector competence in *Frankliniella occidentalis* (Western flower thrips) in the transmission of *Tomato spotted wilt virus*

**DOI:** 10.1002/ece3.2484

**Published:** 2016-10-11

**Authors:** Pamella Akoth Ogada, Thomas Debener, Hans‐Michael Poehling

**Affiliations:** ^1^ Department of Phytomedicine Institute of Horticultural Production Systems Gottfried Wilhelm Leibniz Universität Hannover Hannover Germany; ^2^ Department of Molecular Plant Breeding Institute for Plant Genetics Gottfried Wilhelm Leibniz Universität Hannover Hannover Germany

**Keywords:** *Frankliniella occidentalis*, haplodiploidy, inheritance, intraspecific variation, *Tomato spotted wilt virus*, *tospovirus*, vector competence

## Abstract

The complexity of *tospovirus*–vector–host plant interaction is linked to a range of factors influencing vector's efficacy in virus transmission, leading to high variability in the transmission efficiency within vector populations. Main shortcomings of most studies are the missing information on the intrinsic potential of individual insects to serve as efficient vectors, both at phenotypic and at genotypic levels. Moreover, detailed analysis of vector competence heredity and monitoring the splitting of both genotypes and phenotypes in filial generations has not been reported. In this study, using the model system *Frankliniella occidentalis* and *Tomato spotted wilt virus*, we evaluated the inheritance and stability of the trait vector competence in a population through basic crossings of individually characterized partners, as well as virgin reproduction. We hypothesized that the trait is heritable in *F. occidentalis* and is controlled by a recessive allele. From the results, 83% and 94% of competent and noncompetent males respectively, inherited their status from their mothers. The trait was only expressed when females were homozygous for the corresponding allele. Furthermore, the allele frequencies were different between males and females, and the competent allele had the highest frequency in the population. These suggest that the trait vector competence is inherited in single recessive gene in *F. occidentalis*, for which the phenotype is determined by the haplodiploid mechanism. These findings are fundamental for our understanding of the temporal and spatial variability within vector populations with respect to the trait vector competence and at the same time offer an essential basis for further molecular studies.

## 1 Introduction

The order *Thysanoptera* encompasses about 7,700 described species (CSIRO Australia, 2005), out of which only about 14 species have been identified as vectors of tospoviruses (Riley, Joseph, Srinivasan, & Diffie, 2011), indicative of the specificity of these virus–vector interactions. All the reported vector species belong to the subfamily *Thripinae* of the family *Thripidae* (Mound, 2002). *Frankliniella occidentalis* (Pergande) (Western flower thrips) is considered the most economically important pest among thysanopterans causing enormous annual economic losses worldwide (Kirk, 2002) both by direct damage and competence to transmit five of the 14 recognized *tospovirus* species (Whitfield, Ullman, & German, 2005). It is reported to be the most efficient vector of *Tomato spotted wilt virus* (TSWV) (Whitfield et al., 2005), which currently ranks among the top ten most economically important plant viruses worldwide (Parrella, Gognalons, Gebre‐Selassie, Vovlas, & Marchoux, 2003; Scholthof et al., 2011; Sherwood, German, Moyer, Ullman, & Whitfield, 2000) causing serious losses in a wide range of crops and flowers all over the world (Goldbach & Peters, 1994). However, there is interspecific as well intraspecific variation in the capability to vector‐specific tospoviruses which has been associated with rapid coevolution between thrips and tospoviruses (Nagata, Almeida, Resende, De, & De Ávila, 2004). For instance, transmission of TSWV differs considerably between and within populations of *F. occidentalis* (van de Wetering, van der Hoek, Goldbach, Mollema, & Peters, 1999) as well as in *Thrips tabaci* (Lindeman) (Cabrera‐La Rosa & Kennedy, 2007; Jacobson, Booth, Vargo, & Kennedy, 2013; Westmore, Poke, Allen, & Wilson, 2013).

The transmission of TSWV to healthy plants by *F. occidentalis* follows a persistent and propagative manner, mainly by the adults during feeding (Sherwood et al., 2000; Whitfield et al., 2005), but adults are only efficient vectors if the acquisition of the virus occurs at the early larval stages (Moritz, Kumm, & Mound, 2004; Whitfield et al., 2005), followed by replication inside the host midgut, passing the midgut barrier and uptake and multiplication in the salivary glands during thrips development (Nagata, Inoue‐Nagata, Smid, Goldbach, & Peters, 1999). The pronounced change in virus acquisition during thrips ontogeny has been related to a temporary displacement of the thrips' brain into the prothoracic region in the early developmental stages, which leads to casual association of the midgut and the salivary gland, enabling the flow of the virus between these two chambers (Moritz et al., 2004). However, this association is broken as the thrips develops, which explains the age‐specific acquisition/transmission characteristics of this vector. Furthermore, transovarial transmission of TSWV is not possible in *F. occidentalis*, so each generation must re‐acquire the virus for the disease epidemic to continue (Nagata et al., 1999). However, this specificity in the virus transmission cycle can neither explain the always high individual variability in vector competence when same aged L1 thrips are subjected to the same virus sources (*tospovirus* infected host plants), nor the dynamic change of relative amounts of vector competent individuals in populations of different sizes, or in isolation (inbreeding).

Several studies have postulated that the variability in transmission efficacy observed in thrips populations is due to differences in sexes (van de Wetering et al., 1999), or in genotypes, which can be separated for instance by random amplification of polymorphic DNA analysis (Gillings, Rae, Herron, & Beattie, 1996), as well as using mitochondrial cytochrome c oxidase 1 gene sequence analysis (Jacobson et al., 2013; Westmore et al., 2013), indicating that the trait vector competence is manifested in the genome and it is variable. Cabrera‐La Rosa and Kennedy (2007) hypothesized that vector competence trait is recessively inheritable in the case of TSWV and *T. tabaci* Lindeman. Furthermore, Halaweh and Poehling (2009) reported in their preliminary crossing experiments with *Ceratothripoides claratris* (Shumsher) (Thysanoptera: Thripidae) vectoring *Capsicum chlorosis virus* (CaCV) that males inherit the trait vector competence only from their mothers. Additionally, under inbreeding conditions in an isolated colony, the ratio of competent versus noncompetent individuals strongly declined with increasing homozygosity, suggesting also that vector competence in *C. claratris* is controlled by a recessive allele (Halaweh & Poehling, 2009). It is, however, not obvious yet, whether this mechanism of inheritance found in *C. clarathris* and *T*. *tabaci* is common in all thrips–*tospovirus* relationships.

Based on the hypothesis that vector competence of *F. occidentalis* is a heritable trait linked to a recessive allele, we performed inheritance experiments with individually characterized *F. occidentalis* (competent [transmitters] or noncompetent [nontransmitters]) and evaluated the competence status of offspring produced both parthenogenetically by individual virgin females and from controlled crossings of individual partners with determined competence status. The focus on individual crossings is based on our hypotheses that the available inheritance studies on the trait vector competence in *C. clarathris* and *T*. *tabaci* (Cabrera‐La Rosa & Kennedy, 2007; Halaweh & Poehling, 2009) crossing experiments were performed only at population levels, which might have obscured the contribution of individual's genetic constitution.

## 2 Materials and Methods

### 2.1 Host plant

For the study, *Capsicum annuum* L. (Solanaceae) (4–5 leaf stages) was selected as a host plant for *F. occidentalis* as well as TSWV maintenance, and was used in all the experiments. To facilitate propagation of the virus for stock inoculum, *Nicotiana benthamiana* L. (Solanaceae) (3–4 leaf stage) served as a reservoir host plant alongside *C. annuum*, because of its susceptibility to the virus and ease in handling during mechanical inoculation. *Phaseolus vulgaris* L. (Fabaceae) was used for the maintenance of *F. occidentalis* stock culture. All clean host plants were maintained in a thrips proof nursery chamber at glasshouse conditions (28–30°C and 70%–80% r.h.).

### 2.2 Thrips culture and maintenance

*Frankliniella occidentalis* strain (*Fo*2) was obtained from Wageningen University Laboratory of Virology in the Netherlands, and maintained as stock culture on bean plants (*P. vulgaris*) at 2–3 leaf stage, in cages covered with thrips tight gauze in climate cabins at constant conditions (25 ± 2°C; 60–70% r.h.; L16:D8). A synchronized rearing which served as *F. occidentalis* source for all the experiments was established from the stock culture on young fresh green bean pods (*P. vulgaris*), supplemented with commercial honeybee pollen mixture (Naturprodukte‐mv.de; Naturprodukte Lembcke, Faulenrost, Germany) in Plexiglas cages closed on top with thrips proof 64‐μm nylon gauze. Synchrony was achieved by transferring the old bean pods with a cohort of freshly deposited eggs, into new cages for L1 hatching and replacing them with new bean pods for further egg laying, this was performed at one‐day intervals. Isolated rearing of such same aged cohorts ensured the availability of all life stages of *F. occidentalis* at any one time.

### 2.3 TSWV isolate and maintenance

The TSWV isolate (TSWV‐12) was also obtained from Wageningen University Laboratory of Virology in the Netherlands, and confirmed by double‐antibody sandwich enzyme‐linked immunosorbent assay (DAS‐ELISA). The isolate was maintained at glasshouse conditions (28–30°C and 70%–80% r.h.) by series of mechanical inoculations performed after every 2–3 weeks on *C. annuum* (4–5 leaflet stage) and *N. benthamiana* (4–5 leaflet stage). To reduce the chances for defective interference of the virus, feasibly caused after several serial passages by mechanical inoculation, and to ensure viability of the virus for transmission by *F. occidentalis*, fresh inoculations with the original source inoculum were made after every fifth serial passage. Moreover, as a backup, a parallel thrips‐mediated inoculation was maintained on *C. annuum* (4–5 leaflet stage) in an isolated climate chamber (25 ± 2°C, 50%–60% r.h. and L16:D8) in thrips proof cages.

Protocol developed by Mandal, Csinos, Martinez‐Ochoa, and Pappu (2008) was used for the mechanical inoculation. In summary, the inoculum contained TSWV‐infected leaf sap prepared in 0.1 mol/L phosphate buffer, 0.2% sodium sulfite, 0.01 mol/L 2‐mercaptoethanol and 1% each of celite 545, and carborundum 320 grit. A soft finger rubbing technique was used in delivering the inoculum onto the test plants leaves. The inoculated plants were kept under glasshouse conditions. The first symptoms appeared 10–14 days after inoculation, and successful transmission of the virus in systemically infected leaflets was confirmed using DAS‐ELISA, targeting the viral nucleocapsid protein. The infected leaves were then used as the inoculum source for further series of mechanical inoculation as well as for virus acquisition by the newly hatched first‐instar larvae (L1) in the experiments.

### 2.4 Detection of TSWV in host plants by double‐antibody sandwich Enzyme‐linked immunosorbent assay

Double‐antibody sandwich enzyme‐linked immunosorbent assay was used to confirm the observed symptoms on the host plants. Two TSWV‐specific antibodies (polyclonal mixture ex rabbit) (Loewe) were used. A polystyrol‐96‐well microtiter plate (medium binding) was first coated with the antigen‐specific coating antibody (IgG), followed by addition of sap extracted from leaf disks to the coated wells: Samples were extracted in phosphate‐buffered saline with Tween plus polyvinylpyrrolidone (PVP) (Loewe) and egg albumin (Loewe) (PBS‐TPO). The third step involved the addition of the enzyme‐labeled antibody‐AP‐conjugate, forming the double‐antibody sandwich. *p*‐Nitrophenyl phosphate (Loewe) dissolved in a substrate buffer was then added to initiate enzymatic reaction, resulting in yellow colored 4‐nitrophenol as product. All samples and known TSWV‐positive and TSWV‐negative controls were tested in duplicate. Color development was measured in a spectrophotometer at 405 nm using Multiskan FC microplate photometer, from Thermo Scientific. The average value of the noninfected controls from the plants, plus three times their standard deviation, made the minimum threshold for positive ELISA values. Therefore, samples were considered TSWV‐positive if the mean optical density reading exceeded the minimum threshold.

### 2.5 Characterization of individual *F. occidentalis* competence status (biotest–phenotyping)

Newly hatched L1 larvae (<12 hr old) were collected from the synchronized rearing by softly blowing them off the bean pod onto a TSWV‐infected *C. annuum* leaf (high virus titer confirmed by DAS‐ELISA) in a gypsum (CaSO_4_) petri dish (9 cm diameter) for an acquisition access period (AAP) throughout their larval stages until pupation. The gypsum petri dishes were made up of a thin layer of gypsum and charcoal mixture (ratio 9:1) covering the bottom, moistened with a few milliliters of distilled water, and then overlaid by a piece of filter paper to absorb excess water. The lid of each petri dish had three equally spaced holes (12 mm diameter) covered with thrips proof (64‐μm) nylon mesh for ventilation. Once closed, all of the petri dishes were sealed with Parafilm M^®^ (Pechiney Plastic Packaging, Inc., USA) to avoid thrips escape. After 4–6 days, the resulting pupae were individually transferred onto a virus free *C. annuum* leaf disk (17 mm diameter) placed in a new petri dish (6 cm diameter) lined with moist filter papers for adult emergence. The emerging adults were allowed to feed on the virus free leaf disk for 36‐hour inoculation access period (IAP) before being transferred onto a new leaf disk for further experiments (virgin reproduction or crossing, see below). The old leaf disks were incubated for 3 days before being subsequently assayed for successful virus transmission using amplified DAS‐ELISA (an improved form of DAS‐ELISA in terms of sensitivity, at least 10‐fold higher), which amplifies the ELISA signals using amplification kit from Invitrogen Life Technologies GmbH, Cat. No. 19589‐019, following the manufacturer protocol. Based on the results, individual thrips were characterized as either competent or noncompetent virus transmitters. Further confirmation of the individual competence status was performed using the status of their offspring. In both virgin reproduction and basic crossing experiments, the percentage of competent thrips at population level was considered to be the same as the percentage of leaf disks that gave positive readings in the Amplified DAS‐ELISA tests. We presumed that the allele for the trait vector competence is recessive (c) while noncompetence is dominant (C). All the experiments were performed in the climate chambers at 25 ± 2°C, 50%–60% r.h. and L16: D8.

### 2.6 Virgin reproduction and inheritance evaluation

Ten noninseminated female *F. occidentalis* were randomly selected after IAP prior to the characterization step (described above) and individually used as parents in the virgin reproduction (parthenogenetic) experiment. Each virgin female was placed on a healthy leaf disk for egg laying, and the leaf disk was replaced at one‐day interval with a new one for further oviposition. The old leaf disks with cohorts of eggs were individually kept for collecting the newly hatched F1 larvae, which were allowed an AAP on infected *C. annuum* leaves until pupation. Several batches of F1 larvae were collected until the death of the mother thrips. After the AAP, the resulting pupae were individually transferred onto a new virus free leaf disks, and the hatching adults (F1, all males) were tested for successful transmission of the acquired virus. This way, the vector competence of each individual offspring was determined, and the percentage of competent and noncompetent individuals calculated per virgin parent. Additionally, the resulting F1 status was used to distinguish between the homozygous and heterozygous noncompetent parents based on the hypothesized F1 phenotypes per parental status (Table 1). A threshold of 30% was used as follows: that is, if atleast 30% of the resulting offspring from a noncompetent female parent were competent, then the female parent was considered heterozygous for this trait. This experiment was repeated five times.

**Table 1:** The possible parental genotypes virgin females and the resulting F1 outcome, with the expected phenotypes and the possible genotypes

Parental phenotypes (virgin females) (Possible genotypes)	F1 offspring phenotypes (all males) (Possible genotypes)
♀ com (cc)	♂ com (c‐)
♀ n‐com (CC)	♂ n‐com (C‐)
♀ n‐com (Cc)	♂ n‐com:com (½C‐:½c‐)

CC & C‐: homozygous noncompetent female and noncompetent male status, respectively; Cc: heterozygous noncompetent female; cc & c‐: homozygous competent female and competent male status, respectively.

### 2.7 Basic crossing experiments and segregation analysis

Males and noninseminated females selected randomly after the IAP prior to characterization were used as parents in subsequent crosses. The presumed cross‐combinations for the experimental series were based on the hypothesis that the trait vector competence is recessive (c). Table 2 summarizes the hypothesized crosses and the expected offspring status.

**Table 2:** Intended parental cross‐combinations and the expected F1 offspring outcome with the expected phenotypes and the possible genotypes

Cross‐combinations#	Possible parental crosses genotypesFemale (♀) × Male (♂)	Expected F1 offspring phenotypes (genotypes)	
		♀	♂
1	comp (cc) × n‐comp (C‐)	n‐com (Cc)	com (c‐)
2	n‐comp (CC) × comp (c‐)	n‐com (Cc)	n‐com (C‐)
3	n‐comp (Cc) × comp (c‐)	n‐com:com (½Cc:½cc)	n‐com:com (½C‐:½c‐)
4	comp (cc) × comp (c‐)	com (cc)	com (c‐)
5	n‐comp (CC) × n‐comp (C‐)	n‐com (CC)	n‐com (C‐)
6	n‐com (Cc) × n‐comp (C‐)	n‐com (½CC:½Cc)	n‐com:com (½C‐:½c‐)

CC & C‐: homozygous noncompetent female and noncompetent male status, respectively; Cc: heterozygous noncompetent female; cc & c‐: homozygous competent female and competent male status, respectively.

Each cross was made up of a couple consisting of one male and one female *F. occidentalis*, placed together on a single virus free leaf disk to mate and reproduce. The leaf disks were replaced at one‐day interval with new ones for further oviposition until the death of the mother thrips, and the old leaf disks were individually kept for collecting the cohorts of daily hatched F1‐larvae, which were transferred onto TSWV‐infected *C. annuum* leaves for an AAP until pupation. The resulting pupae were individually transferred onto healthy leaf disks, and the emerging adults were tested for the trait vector competence by leaf disk assay (described above). The percentage of competent male and female offspring from each parental cross was calculated and further used to confirm the characterized competence status of their parents. Noncompetent F1 females from all the possible cross‐combinations (Table 2) were allowed to reproduce parthenogenetically in a separate leaf disk assay. The competence status of the resulting offspring (F2 generation, all males) was tested as described to determine the homo/heterozygous specificity of the noncompetent F1 generation females. Eight repeats each consisting of 10 random crosses of the possible six combinations (Table 2) were performed, and ideally, all offspring of individual pairs were tested for vector competence in consideration of the expected stochastic component given, for example, by variability in virus acquisition and transmission independent from the trait vector competence.

### 2.8 Expected allele and genotype frequencies

Hardy–Weinberg equilibrium (HWE) was taken as the basis for analysis of both allele and genotype frequencies. It assumes stable allele and genotype frequencies in a population if there are no evolutionary influences. When assuming random mating in diploid thrips (females), and vector competence as recessive (c) while noncompetence as dominant (C) allele, HWE is given as:

(1) p2+2pq+q2=1(For genotype frequency),

(2) p+q=1(For allele frequency),

where *p* is the frequency of the dominant allele C, *q* is the frequency of the recessive allele c, *p*_2_ = homozygous dominant, *q*_2_ = homozygous recessive, and 2*pq* = heterozygous. Equation (1) was used for the genotype frequencies and (2) for allele frequencies, for the diploid population (females). While for the males, we assumed that the allele frequencies are similar to their mothers, and because they are haploids, Equation (2) was used for both the allele and genotype frequencies. Discrepancy between the observed and the expected frequencies in the test population was evaluated for the fit to the HWE.

### 2.9 Statistical analysis

The classical Mendelian values (assuming single‐gene inheritance) were used to calculate the expected ratios, which were further adjusted using the misclassification probabilities determined from the parental status. Chi‐square analysis was used to test the observed ratios against the expected ratios. Male and female offspring data per cross‐combination were analyzed separately. Parents–offspring relatedness was analyzed using chi‐square test of independence (Fisher's exact test only for small sample numbers), to test whether the observed phenotype frequencies in the offspring were independent from the parental status for the trait vector competence. The expected genotype and allele frequencies of the observed phenotype (biotest) were analyzed and compared using the Equations (1) and (2), and the discrepancy between them evaluated using chi‐square, to determine the HWE status of the population. The chi‐square test was performed using PROC FREQ/chi‐square command in Statistical Analysis System 9.0 for Windows (SAS/STAT Software, 2002), and a significance level of .05 was used.

## 3 Results

### 3.1 Parental competence status represented in the experimental population

**Figure 1:**
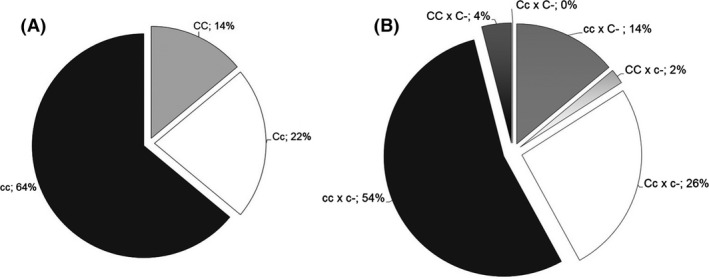
Representation of competence status (genotypes) in the test populations of *Frankliniella occidentalis*; (A) representation of different genotypes in virgin females' population, (B) cross‐combinations representation of different genotypes. CC & C‐: homozygous noncompetent female and noncompetent male status, respectively; Cc: heterozygous noncompetent female; cc & c‐: homozygous competent female and competent male status, respectively

The representation of the three possible genotypes in the entire test population of virgins was uneven according to the random samples taken. About 64% of the population were determined to be homozygous competent (cc), 22% were heterozygous noncompetent (Cc), and 14% were homozygous noncompetent (CC) (Figure 1A). Determinations were based on both the amplified DAS‐ELISA results as well as the offspring status. On the other hand, from the crossing experiments out of the six hypothesized cross‐combinations, only five were achieved, with the highest representation being the combination where the couples were competent (i.e., cc × c‐) at 54%, while a combination of heterozygous noncompetent female and noncompetent male (Cc × C‐) could not be ascertained (Figure 1B).

### 3.2 Evaluation of inheritance of the trait vector competence in virgins' reproduction

**Figure 2:**
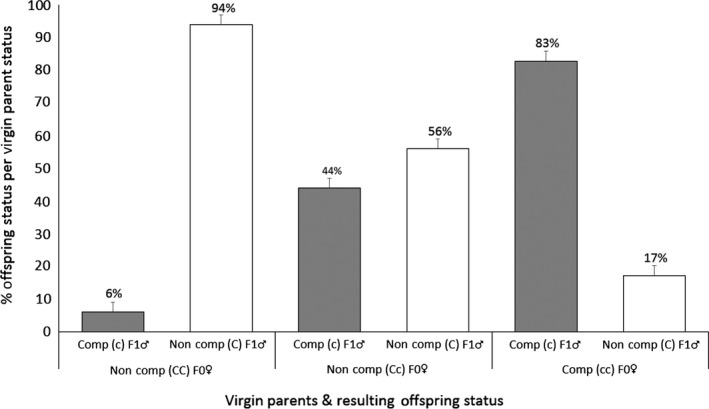
Evaluation of inheritance of the trait vector competence by *Frankliniella occidentalis*: Status of progeny from virgin females. F0♀ represents the virgin female parents, and F1♂ represents the male offspring. Comp = competent status, non‐comp = noncompetent. CC & C‐: homozygous noncompetent female and noncompetent male status, respectively; Cc: heterozygous noncompetent female; cc & c‐: homozygous competent female and competent male status, respectively

Parthenogenetic reproduction by the virgin females resulted in 100% male F1 offspring. Of the progeny from the homozygous noncompetent virgin females, 94% of the F1 were noncompetent, while from the heterozygous noncompetent virgin parents, 56% of the F1 were noncompetent and 44% were competent. From the competent parents, 83% of the resulting F1 were competent and 17% were noncompetent (Figure 2). The competence status of the resulting offspring significantly related to the status of their mothers, χ²(*df* = 2, *N* = 397) = 123.34, *p *<* *.0001.

### 3.3 Basic crossings—analysis of inheritance of the trait vector competence in F1

We considered the total number of F1 per cross‐combination in all the repeats for our analysis, as the offspring status of similar crosses was comparable. Cross‐combinations involving a homozygous noncompetent female parent (CC) resulted in 100% noncompetent male and female offspring (Table 3: combinations number 2 [χ²(*df *= 1, *N* = 27) = 0, *p *=* *1] and 5 [χ²(*df* = 1, *N* = 17) = 0, *p *=* *1]), regardless whether the male parent was competent (c‐) or not (C‐). Crossing of competent females (cc) with noncompetent males (C‐) resulted in 100% noncompetent female offspring and 89% competent male offspring (Table 3: combination number 1 [χ²(*df* = 1, *N* = 52) = 36.98, *p *<* *.0001]). A cross of competent parents (cc × c‐) resulted in 87% female offspring being competent (cc), and 84% of male offspring being competent (c‐) (Table 3: combination number 4 [χ²(*df* = 1, *N* = 289) = 0.51, *p *=* *.48]). In the cross‐combination involving a heterozygous noncompetent female (Cc) and a competent male (c‐), the status of the female parent was determined as being heterozygous noncompetent (Cc) indirectly based on the resulting percentages of the offspring status; approximately 57% and 44% of F1 females and males, respectively, were competent (Table 3: combination number 3 [χ²(*df* = 1, *N* = 119) = 1.83, *p *=* *.176]). However, it was not possible to find cross‐combination number 6 (Cc × C‐) (Table 3) in our test population. When further evaluating the F2 of the noncompetent virgin F1 females from the crosses, 100% of the resulting F2 males from the cross‐combinations 4 and 5 (see Table 3) were competent (c‐) and noncompetent (C‐), respectively, indicating homozygosity of the F1 virgin parents. For the cross‐combinations 1, 2, and 3, the resulting competence status of the F2 was approximately 50% of both competent (c‐) and noncompetent (C‐) individuals, indicating heterozygosity of the virgin F1 females in these combinations.

**Table 3:** The relative numbers of the resulting offspring (F1) competent and noncompetent per parental cross‐combination

The relative number of competent and noncompetent F1 per parents cross‐combination							
No.	Possible parental crosses phenotypesFemale (♀) × Male (♂)	Competent F1			Noncompetent F1		
		♀	♂	Total	♀	♂	Total
1	comp (cc) × n‐comp (C‐)	0	32	32	16	14	20
2	n‐comp (CC) × comp (c‐)	0	0	0	11	16	27
3	n‐comp (Cc) × comp (c‐)	24	34	58	18	43	61
4	comp (cc) × comp (c‐)	100	146	246	15	28	43
5	n‐comp (CC) × n‐comp (C‐)	0	0	0	8	9	17
6	n‐com (Cc) × n‐comp (C‐)	0	0	0	0	0	0
	Total	124	212	336	68	100	146

### 3.4 Analysis of relatedness based on the trait vector competence

Parents–offspring relatedness was evaluated using chi‐square analysis. We found high significance between mother–sons, mother–daughters, and father–daughters' relations. However, there was no relation whatsoever between father–sons (χ^2^(*df* = 1, *N* = 301) = 0.0117, *p *=* *.9138, Table 4). This calculation was based on the inheritance probability of an allele by the offspring from the parents.

**Table 4:** Chi‐square analysis of the parental–offspring relatedness

Relations	χ^2^	*df*	*p*	*N*
Mother <=> Sons	79.58	2	<.0001	301
Mother <=> Daughters	23.64	2	<.0001	181
Father <=> Sons	0.0117	1	.9138	301
Father <=> Daughters	60.19	1	<.0001	181

Further evaluation of the male F2 from the F1 of virgin reproduction showed a relationship between grandmother and grandson, as well as between grandfather and grandson (results not shown).

### 3.5 Expected genotype and allele frequencies

The allele and genotype frequencies of the virgin female parents (diploids) were determined based on the HWE formulas (1) and (2). We assumed that the males' allele frequencies were similar to their mothers. The expected frequency of the competent alleles (c) in both males and females was higher (0.8) compared to the noncompetent alleles (C) (0.2). From the determined allele frequencies, the expected proportions of genotypes within our test population were evaluated for both males and females based on the described formulas. The expected genotype frequencies in males were similar to their allele frequencies because they are haploids. In females, the homozygous competent genotype had the highest frequency (0.64), followed by the heterozygous noncompetent (0.32), while the homozygous noncompetent genotype had the lowest frequency (0.04) (Table 5).

**Table 5:** Expected proportions of genotypes for the trait vector competence in both males and females in the test population

Males		Females	
(Genotypes (**♂**)	Expected genotype frequencies	Genotypes (♀)	Expected genotype frequencies
C‐	*p *=* *0.2	CC	*p* ^2^ = 0.04
c‐	*q *=* *0.8	Cc	2*pq* = 0.32
		cc	*q* ^2^ = 0.64

In the analysis of deviation from the HWE, the gene frequencies in the samples were first calculated from the observed numbers using the Equation (2); the observed numbers in the sample population are presented in Table 6. Chi‐square test comparison between the observed and the expected numbers showed low amount of heterozygotes but an excess of competent (recessive) homozygotes in the diploid females. The differences between the observed and the expected numbers were of low significance (*p *=* *.0382) in females, while in males, the difference was highly significant (*p *<* *.0001). Evaluation of the difference in the population as a whole (both males and females) was also highly significant (*p *<* *.0001) (Table 6), which is an indication of deviation from the HWE. As the allele frequencies were estimated from the same data, chi‐square test has only one degree of freedom, to ensure that the observed and the expected numbers agree both in their allele frequencies and in their totals. From the observed genotype numbers in the test population, we calculated the allele frequencies using the HWE formulas. Females had higher frequencies of the competent allele (c) (0.841) compared to males (0.680) (Table 7).

**Table 6:** Chi‐square analysis for the agreement with the Hardy–Weinberg equilibrium using the observed and the expected numbers of genotypes in the test population

		Females genotypes				Males genotypes		
		CC	Cc	cc	Total	C‐	c‐	Total
Numbers observed		8	45	139	192	100	212	312
Numbers expected		7.68	61.44	122.88	192	62.4	249.6	312
Females	χ^2^(1,192) = 6.53	*p *=* *.0382						
Males	χ^2^(1,312) = 88.06	*p *<* *.0001						
Total	χ^2^(1,504) = 94.58	*p *<* *.0001						

**Table 7:** Allele frequencies calculated from the observed numbers in the test population

	Number of alleles in the population			Frequency of the competent allele (c)*q*
	C	c	Total	
In females	61	323	384	0.841
In males	100	212	312	0.680

With the observed high allele and genotype frequencies for the competent trait, the same data were used to evaluate the frequencies for the listed possible cross‐combinations (Table 8) within a hypothesized one million pairs of parents. The results show that a cross‐combination with both parents having the competent trait (cc × c) will have the highest frequency (0.512) within the population. On the other hand, the cross‐combination that will have the lowest frequency of occurrence in the population (0.008) is the one with both parents being noncompetent (CC × ‐C) (Table 8).

**Table 8:** Estimation of the expected allele frequencies for the hypothesized cross‐combination per million pairs of parents

Mating types	Expected frequencies		
	Proportions	%	Per million pairs of parents
CC × ‐c	*p* ^2^ *q* = 0.032	3.20	32,000
cc × ‐C	*pq* ^2^ = 0.128	12.80	128,000
Cc × ‐C	2*p* ^2^ *q* = 0.064	6.40	64,000
Cc × ‐c	2*pq* ^2^ = 0.256	25.60	256,000
cc × ‐c	*q* ^3^ = 0.512	51.20	512,000
CC × ‐C	*p* ^3^ = 0.008	0.80	8,000
Totals	1	100	1,000,000

## 4 Discussion

Sex determination in *F. occidentalis* is by haplodiploid mechanism, with females laying two kinds of eggs; fertilized eggs that have diploid sets of chromosomes from both parents developing to females, whereas unfertilized eggs with only one copy (haploid) of the mother's chromosomes produce males (Crespi, Evolution, & Mar, 1991; Hedrick & Parker, 1997). Therefore, females have twice as many copies of alleles compared to males, which is an important factor in the determination of the overall allelic frequencies in a population (Hedrick & Parker, 1997). Haplodiploid insects are reported to have lower levels of heterozygosity compared to the diploid insects and hence lower levels of genetic variability (Crespi et al., 1991). This could partly explain the high percentage of individuals bearing recessive allele for the trait vector competence (c) observed in our test population and also the low and even complete lack of certain cross‐combinations. Reduced heterozygosity is also predicted in X‐linked genetic systems, which are very similar to the haplodiploid systems (Hedrick & Parker, 1997). Furthermore, the observed reduction in heterozygosity could be due to evolutionary selection by fitness processes in our test population (Li, 1976), because the samples (cohorts) were obtained from a stock culture which had been maintained for a long time in isolated rearing.

From this study, we can strongly infer that the trait vector competence in *F. occidentalis* is inherited in a haplodiploid recessive single‐gene (Mendelian) pattern, which is similar to the X‐linked recessive inheritance pattern (Falconer & Mackay, 1996; Hedrick & Parker, 1997). This inheritance pattern was obvious from the virgin reproduction results, where all offspring were males (as expected) and inherited their competence status from their mothers. This result confirms earlier findings by Halaweh and Poehling (2009), who reported a vertical inheritance of the trait vector competence from the mothers to their male offspring in *C. claratis* when studying the transmission of CaCV. Moreover, by analyzing relatedness between parents and offspring in the crossing experiment, we found no relation of male offspring to their fathers with regard to inheritance of the trait vector competence, which confirms the above characterized pattern of inheritance. Additionally, we were able to examine the mode of inheritance by analyzing F2 males from the virgin F1 females in the crossing experiment, eliminating the need for backcrossing or crossing F1 males and females as commonly performed with diploid species (Crowder et al., 2009). The results confirmed the recessive nature of the trait vector competence because it was only manifested in females (diploids) that were homozygous for this allele, which was obvious from the ~100% of their offspring being competent. However, the 30% competent F1 used as the cutoff in the determination of the hetero/homozygous noncompetence status of the female parents could have led to the observed 6% competent offspring in the homozygous noncompetent parents, where 100% noncompetent offspring was expected.

Recessive traits are often associated with deleterious effects (characterized by selective elimination of homozygotes) in most X‐linked and haplodiploid inheritance patterns (Borgia, 1980). However, our study revealed the contrary. In this system of haplodiploids with the specifics of TSWV‐*F. occidentalis* relationship, we found strong indications for an increase in the frequency of this trait over time, leading to homozygosity biased toward the recessive allele in the population. We could therefore presume that the trait vector competence is favorable and that the increase in frequency of the recessive allele (competent) over time is due to the relatively small selective effects and dosage compensation by equalization of gene expression in the haplodiploids. This contradicts earlier reports by Halaweh and Poehling (2009), and Cabrera‐La Rosa and Kennedy (2007), who observed an increase in homozygosity of the dominant allele when investigating inheritance of vector competence in *C. claratris* and *T. tabaci*, in the transmission of CaCV and TSWV, respectively, linking it to inbreeding effects. They both used populations in their crossing experiments and therefore could have overlooked the important contribution of individual insect genetics. Furthermore, Hedrick and Parker (1997) also reported a lower inbreeding coefficient in haplodiploid or X‐linked genes compared to autosomal genes, because males are haploid (zero inbreeding coefficient) and therefore cannot be inbred.

Apart from individual genetics, the observed increase in frequency of the competent allele could also be associated with the hypothesis that exposure of *F. occidentalis* to TSWV triggers the vectors' immune response resulting in improved fitness in terms of survival and longevity (de Medeiros, Resende, & de Avila, 2004; Ogada, Maiss, & Poehling, 2013). On the other hand, prolonged feeding of thrips on virus‐infected plants has been reported to have deleterious (pathological) effects to the vectors, presumably due to high virus load and virus propagation in the vector′s body which could overpower the initially induced immune system (Shalileh, Ogada, & Poehling, 2016). However, reports are also emerging indicating vector manipulation by the virus in terms of preferential behavior, which favors the multiplication and the spread of the virus and at the same time promoting longer survival of the vector (fitness) (Shalileh et al., 2016), presumably blinding off the expected deleterious effects on the vector. Improved fitness is also reported in bees (Hymenoptera), as a result of the haplodiploid sex determination system due to indirect selection (Foster & Ratnieks, 2001).

The evaluation of the expected allele frequencies for the haplodiploid females and males follows the estimation of alleles in diploid and haploid individuals, respectively (Li, 1976). Studies have shown that if there is a difference in the initial frequencies between the two sexes with no overlapping of the generations, oscillation in the allele frequencies will occur between the two sexes, above and below the average frequency, but the difference is halved in successive generations and the population rapidly approaches an equilibrium with equal frequencies in both sexes (Hedrick & Parker, 1997; Li, 1976). Such differences are already obvious in our analysis of the observed allele frequency between the two sexes. As males receive their entire chromosomes from their mothers, we assumed therefore, that their allele frequencies are identical to their mothers (Cabrera‐La Rosa & Kennedy, 2007; Halaweh & Poehling, 2009; Hedrick & Parker, 1997; Moritz, 1997), and employed HWE for the analysis of genotype and allele frequency. Furthermore, by variation of specific allele frequencies due to the haplodiploid inheritance system (Moritz, 1997), phenotypic variation (vector competence) in subsequent generation can be expected (Gillings et al., 1996; van de Wetering et al., 1999). Therefore, Mendel's law of segregation and the HWE cannot be applied in the same way for both genders, and additionally, the ideal conditions required for the HWE (Crow, 1999) are not adhered to in our experiments. Other factors like the small population size of the parental genotypes used could have brought along high sampling variation in gene frequencies in the successive generation. Moreover, different genotypes in the parental generation may have had different fertilities, which we did not control, and hence may have influenced the allele frequency over time (selection). Additionally, controlled mating in this experiment might have also contributed to the observed allele frequencies.

When discussing the mechanism of vector competence, steps under genetic control and targets for genetic variability are crucial factors. The virus recognition and infection process which involves key proteins (gene products) should be considered. Evidence exists of involvement of viral glycoprotein as determinants in the recognition process in the vector's midgut (Whitfield et al., 2008) and of receptor‐based endocytosis of the virus during its entry via the midgut epithelial cell (Bandla, Campbell, Ullman, & Sherwood, 1998; Kikkert et al., 1998). For interaction with the vector, the involvement of TSWV nonstructural protein (NSs), the gene silencing suppressor in plants, is reported (Bucher, Sijen, De Haan, Goldbach, & Prins, 2003; Ding, Li, Lu, Li, & Li, 2004). Moreover, identification of molecules that might influence the pathogen development within the insect tissues, and the isolation of genes that encode for these molecules, is another approach to clarify the mechanisms controlling *F. occidentalis* vector competence (Beerntsen, James, & Christensen, 2000). Primary immune components are found within the hemolymph and are involved in virus recognition and initiation of defense response (Paskewitz & Christensen, 1996). This mechanism of vector defense and the strategies used by the virus to escape recognition and destruction by the vector′s immune system could be the other important determinant of vector competence, because in the noncompetent vectors, despite the virus particles successfully passing the midgut barrier, they fail to further propagate or are destroyed by the vector's defense response (Beerntsen et al., 2000). Additionally, the genetic makeup of the virus and the host plant can also play a role in the adaptability success of the pathogen within the vector and thus influencing vector competence (de Oliveira Resende et al., 1992; Jacobson et al., 2013; Montero‐Astúa et al., 2014).

Another important aspect in allele frequencies consideration of the trait vector competence in *F. occidentalis* is the difference between males and females in terms of feeding behavior, survival time, and transmission efficiency of the virus (Ogada & Poehling, 2015), which have also been reported to contribute to the transmission variability, as well as influencing virus spread. Therefore, the ratio of males and females in terms of the vector population composition is quite important in this regard (Inoue & Sakurai, 2006).

We can therefore conclude that the genetic makeup of individuals within a vector species population to a larger extent is the main determinant of the success of the specific virus–vector interaction with regard to vector competence, considering that this trait is not present in all individuals (Cabrera‐La Rosa & Kennedy, 2007; Halaweh & Poehling, 2009). Therefore, studying the inheritance of the trait and the behavior of the alleles in the filial generation is an important contribution to a more general understanding of this phenomenon. From an application point of view, the projected increase in allele frequency of the trait vector competence in consecutive generation may partly explain the often aggressive spread of TSWV in crop stands and should be considered when developing control strategies, for instance, predictive models.

This work lays a fundamental foundation in the development of molecular markers for the trait vector competence which would enable a more precise analysis of the trait's behavior overtime, using molecular tools like microsatellites or AFLP. Additionally, development of microsatellite genetic linkage map would enable the assessment of the genetic background controlling vector competence of *F. occidentalis* in the transmission of TSWV. The identification of the molecular marker loci linked to genes that influence vector competence would provide the necessary starting point for map‐based cloning and furthermore the realization of linking molecular and bioassay data.

## Conflict of Interest

None declared.
